# Supplementary material for: A Critical View of the Application of the APEX Software (Aqueous Photochemistry of Environmentally-Occurring Xenobiotics) to Predict Photoreaction Kinetics in Surface Freshwaters
Source: Molecules. 2019 Dec 18;25(1):9. doi: 10.3390/molecules25010009 (PMC7017383; doi:10.3390/molecules25010009)
Supplement: Supplementary file 1 [file molecules-25-00009-s001.zip › 3.2.4_gcc-4.4.0.zip!/3.2.4_gcc-4.4.0/3.2.4_gcc-4.4.0/doc/octave/html/interpreter/Comparing-Strings.html]

Comparing Strings - Untitled


Next: Manipulating Strings,
Previous: Creating Strings,
Up: Strings


---

### 5.4 Comparing Strings

Since a string is a character array, comparisons between strings work
element by element as the following example shows:

```
     GNU = "GNU's Not UNIX";
     spaces = (GNU == " ")
          => spaces =
            0   0   0   0   0   1   0   0   0   1   0   0   0   0
```

To determine if two strings are identical it is necessary to use the
`strcmp` function. It compares complete strings and is case
sensitive. `strncmp` compares only the first `N` characters (with
`N` given as a parameter). `strcmpi` and `strncmpi` are the
corresponding functions for case-insensitive comparison.

— Built-in Function: **strcmp** (s1, s2)  
> Return 1 if the character strings s1 and s2 are the same,
> and 0 otherwise.
>
> If either s1 or s2 is a cell array of strings, then an array
> of the same size is returned, containing the values described above for
> every member of the cell array. The other argument may also be a cell
> array of strings (of the same size or with only one element), char matrix
> or character string.
>
> **Caution:** For compatibility with matlab, Octave's strcmp
> function returns 1 if the character strings are equal, and 0 otherwise.
> This is just the opposite of the corresponding C library function.
>
>
>
> **See also:** strcmpi, strncmp, strncmpi.

— Built-in Function: **strncmp** (s1, s2, n)  
> Return 1 if the first n characters of strings s1 and s2 are the same,
> and 0 otherwise.
>
> ```
>           strncmp ("abce", "abcd", 3)
>                => 1
> ```
>
> If either s1 or s2 is a cell array of strings, then an array
> of the same size is returned, containing the values described above for
> every member of the cell array. The other argument may also be a cell
> array of strings (of the same size or with only one element), char matrix
> or character string.
>
> ```
>           strncmp ("abce", {"abcd", "bca", "abc"}, 3)
>                => [1, 0, 1]
> ```
>
> **Caution:** For compatibility with matlab, Octave's strncmp
> function returns 1 if the character strings are equal, and 0 otherwise.
> This is just the opposite of the corresponding C library function.
>
>
>
> **See also:** strncmpi, strcmp, strcmpi.

— Function File: **strcmpi** (s1, s2)  
> Ignoring case, return 1 if the character strings (or character
> arrays) s1 and s2 are the same, and 0 otherwise.
>
> If either s1 or s2 is a cell array of strings, then an array
> of the same size is returned, containing the values described above for
> every member of the cell array. The other argument may also be a cell
> array of strings (of the same size or with only one element), char matrix
> or character string.
>
> **Caution:** For compatibility with matlab, Octave's strcmpi
> function returns 1 if the character strings are equal, and 0 otherwise.
> This is just the opposite of the corresponding C library function.
>
>
>
> **See also:** strcmp, strncmp, strncmpi.

— Function File: **strncmpi** (s1, s2, n)  
> Ignoring case, return 1 if the first n characters of character
> strings (or character arrays) s1 and s2 are the same, and
> 0 otherwise.
>
> If either s1 or s2 is a cell array of strings, then an array
> of the same size is returned, containing the values described above for
> every member of the cell array. The other argument may also be a cell
> array of strings (of the same size or with only one element), char matrix
> or character string.
>
> **Caution:** For compatibility with matlab, Octave's strncmpi
> function returns 1 if the character strings are equal, and 0 otherwise.
> This is just the opposite of the corresponding C library function.
>
>
>
> **See also:** strcmp, strcmpi, strncmp.

— Function File: validstr = **validatestring** (str, strarray)  
— Function File: validstr = **validatestring** (str, strarray, funcname)  
— Function File: validstr = **validatestring** (str, strarray, funcname, varname)  
— Function File: validstr = **validatestring** (..., position)  
> Verify that str is a string or substring of an element of
> strarray.
>
> str is a character string to be tested, and strarray is a
> cellstr of valid values. validstr will be the validated form
> of str where validation is defined as str being a member
> or substring of validstr. If str is a substring of
> validstr and there are multiple matches, the shortest match
> will be returned if all matches are substrings of each other, and an
> error will be raised if the matches are not substrings of each other.
>
> All comparisons are case insensitive.
>
>
>
> **See also:** strcmp, strcmpi.
